# Development of a core outcome set for effectiveness trials aimed at optimising prescribing in older adults in care homes

**DOI:** 10.1186/s13063-017-1915-6

**Published:** 2017-04-12

**Authors:** Anna N. Millar, Amrit Daffu-O’Reilly, Carmel M. Hughes, David P. Alldred, Garry Barton, Christine M. Bond, James A. Desborough, Phyo K. Myint, Richard Holland, Fiona M. Poland, David Wright, On behalf of the CHIPPS Team, University of East Anglia, Annie Blyth, Kate Massey, Vivienne Maskrey, Clare Symms, Arnold Zermansky

**Affiliations:** 1 School of Pharmacy, Queen’s University Belfast, Belfast, UK; 2 School of Healthcare, Baines Wing, University of Leeds, Leeds, UK; 3 Norwich Medical School, University of East Anglia, Norwich, NR4 7TJ UK; 4 Institute of Applied Health Sciences, School of Medicine, University of Aberdeen, Aberdeen, UK; 5 School of Pharmacy, University of East Anglia, Norwich, UK; 6 School of Health Sciences, University of East Anglia, Norwich, UK

**Keywords:** Core outcome set, COS, Optimising prescribing, Medicines Optimisation, Older adults, Care homes, Delphi technique, Consensus, CHIPPS

## Abstract

**Background:**

Prescribing medicines for older adults in care homes is known to be sub-optimal. Whilst trials testing interventions to optimise prescribing in this setting have been published, heterogeneity in outcome reporting has hindered comparison of interventions, thus limiting evidence synthesis. The aim of this study was to develop a core outcome set (COS), a list of outcomes which should be measured and reported, as a minimum, for all effectiveness trials involving optimising prescribing in care homes. The COS was developed as part of the Care Homes Independent Pharmacist Prescribing Study (CHIPPS).

**Methods:**

A long-list of outcomes was identified through a review of published literature and stakeholder input. Outcomes were reviewed and refined prior to entering a two-round online Delphi exercise and then distributed via a web link to the CHIPPS Management Team, a multidisciplinary team including pharmacists, doctors and Patient Public Involvement representatives (amongst others), who comprised the Delphi panel. The Delphi panellists (*n* = 19) rated the importance of outcomes on a 9-point Likert scale from 1 (not important) to 9 (critically important). Consensus for an outcome being included in the COS was defined as ≥70% participants scoring 7–9 and <15% scoring 1–3. Exclusion was defined as ≥70% scoring 1–3 and <15% 7–9. Individual and group scores were fed back to participants alongside the second questionnaire round, which included outcomes for which no consensus had been achieved.

**Results:**

A long-list of 63 potential outcomes was identified. Refinement of this long-list of outcomes resulted in 29 outcomes, which were included in the Delphi questionnaire (round 1). Following both rounds of the Delphi exercise, 13 outcomes (organised into seven overarching domains: medication appropriateness, adverse drug events, prescribing errors, falls, quality of life, all-cause mortality and admissions to hospital (and associated costs)) met the criteria for inclusion in the final COS.

**Conclusions:**

We have developed a COS for effectiveness trials aimed at optimising prescribing in older adults in care homes using robust methodology. Widespread adoption of this COS will facilitate evidence synthesis between trials. Future work should focus on evaluating appropriate tools for these key outcomes to further reduce heterogeneity in outcome measurement in this context.

## Background

There is an increasing need and expectation for researchers to incorporate and report core outcome sets (COSs) when conducting and evaluating effectiveness trials in healthcare [1]. A COS is a list of outcomes which should be measured and reported, as a minimum, in all effectiveness trials pertaining to a specific health area, thereby facilitating comparisons of outcomes between studies and evidence synthesis [2]. The agreed COS should have relevance and be informative to policy makers and stakeholders alike [3]. The relevance of much well-intentioned and robustly conducted research is limited by extremely varied choices of outcomes, thereby impeding understanding, limiting research synthesis [3] and causing ‘avoidable waste’ [2].

The value of health research is greatly increased when reporting outcomes which are usable, homogeneous and meaningful, and which are therefore also more easily accessed by others. In addition to the reporting of numerous and varied outcomes, a lack of standardised outcomes can lead to reporting bias, i.e. selecting statistically significant results only, or reporting sub-sets of results, which can negatively affect the quality of systematic and meta-analytic reviews [1, 3, 4]. The reporting of outcomes requires both careful selection and appropriate reporting. For example, Hirsch and colleagues [5] identified that more than 25,000 outcomes were reported just once or twice in oncology trials. Furthermore, the Outcome Reporting Bias in Trials (ORBIT) study revealed that more than half of the studies included for review (55%) failed to report complete results for the primary outcome [6]. Thus, the need to obtain consensus on key outcomes is considerable and equally urgent.

The impetus to develop coherent COSs for health research to overcome the problems just described above has largely originated from the COMET (Core Outcome Measures in Effectiveness Trials, 2010) Initiative. COMET ‘brings together researchers interested in the development and application of agreed standardised sets of outcomes’ [7]. COSs are developed through a variety of methods, namely semi-structured discussion, unstructured group discussion, the Delphi technique, Consensus Development Conferences and surveys [2]. To date, COSs have been developed for many areas of health research, including prostate cancer [8], HIV/AIDS [9], lower back pain [10], schizophrenia and bipolar disorder [11], cardio-thoracic surgery [12], asthma [13] and, more recently, pain therapy [14] and falls [15], to name some examples.

The specific focus of this paper is the development of a COS for trials involving interventions to optimise prescribing in older adults in care homes. Medicines optimisation, a term describing the safe and effective use of medicines, has become a significant global public health issue [16]. The global population is rapidly ageing, and in the UK, it has been estimated that almost a quarter of the population will be aged ≥65 years by 2034 [17]. In the UK, the prescription of medicines is the most frequent patient-level healthcare intervention [18], and 60% of all dispensed medicines are prescribed for those aged ≥60 years [19]. The volume of research focussing on medicines optimisation is steadily growing, particularly within hospitals, outpatient settings and primary care [20]. However, there is a relative lack of research conducted within the care home setting. In the UK, 3.2% of people aged 65 and older reside in care homes, and people aged 85 and older represent nearly 60% of the care home population [21]. Moreover, prescribing for older people is complex, and most of the evidence shows that prescribing for older residents in care homes is sub-optimal [22]. An age-associated reduction in physiological capacity alongside an increased prevalence of multimorbidity and polypharmacy contribute to the complexities of prescribing in older adults [23]. Prescribing interventions are the most common interventions that take place in care homes and often target inappropriate prescribing (under-, over-, or mis-prescribing), adverse events and compliance [24], and commonly aim to ‘optimise’ the use of medicines.

Some of the factors contributing to all aspects of poor medicines use in care homes are polypharmacy (care home residents are prescribed an average of eight medicines), inappropriate prescribing, inadequate communication and handover protocols between staff, interruptions during drug rounds, inadequate communication across the various healthcare interfaces’ and the lack of clear responsibility for the review of patients’ medicines [24]. To illustrate further, Loganathan and colleagues’ systematic review [20] of interventions to optimise prescribing in care homes reported heterogeneity in outcomes, hindering the development of firm conclusions and recommendations for practice. Additionally, a recently published Cochrane Review conducted by Alldred and colleagues [22] of the effectiveness of interventions to optimise prescribing for older people living in care homes found that, whilst there was some evidence of improvements in medicines optimisation as a result of interventions, firm conclusions relating to the overall effectiveness of interventions were difficult to draw due to, in part, the heterogeneity in outcomes measured across studies. The overall impact of interventions aimed at optimising prescribing to improve outcomes for care home residents, therefore, remains unclear, and the overall value of research remains limited. Clearly, a more cohesive and standardised approach is needed to improve the quality of evidence syntheses to help develop reliable conclusions which can, in turn, influence both policy and practice.

The purpose of this paper is to describe the process of developing a COS for use in effectiveness trials aimed at optimising prescribing in older adults in care homes. This COS was developed within a wider programme of study — the Care Homes Independent Pharmacist Prescribing Study (CHIPPS) (https://www.uea.ac.uk/chipps) – a UK programme grant which incorporates a multicentre cluster-randomised controlled trial to determine the effectiveness and cost-effectiveness of pharmacist independent prescribers taking responsibility for the prescribing of patients’ medicines in care homes. The scope of the COS was informed primarily by the needs of the wider CHIPPS research programme. The aim and scope of the present study were therefore to develop a COS applicable to effectiveness trials, involving any intervention type, to target optimising prescribing in older adults in care homes.

## Methods

The study was prospectively registered with the COMET Initiative (registration number: 843 available online at: http://www.comet-initiative.org/studies/details/843). Development of the COS involved two successive phases which were informed primarily by the published guidance from Williamson et al. [1] on COS development methodology. The first phase involved generating a long-list of outcomes for consideration for inclusion in the COS, through both a review of the published literature and stakeholder involvement (see below). The second phase utilised the Delphi technique [13], in the form of a web-based questionnaire, to elicit consensus on the final list of outcomes to be included in the COS.

### Phase 1: Generating and refining the long-list of outcomes

The aim of this phase was to identify potential outcomes for inclusion in the COS. As such, we sought to identify only ‘what’ to measure, not ‘how’ outcomes could be measured (i.e. the identification of different measurement instruments used to measure the same outcome). To generate the long-list of potential outcomes to be considered for inclusion in the COS, two parallel strategies were employed. The first strategy involved identifying outcomes through a review of the published literature relating to interventions to optimise prescribing in care homes. For the purposes and intended scope of this COS, ‘care homes’ were defined as nursing homes, residential care homes, skilled-nursing facilities, assisted-living facilities and aged-care facilities. The studies included in the recently updated Cochrane Review [22] of interventions to optimise prescribing in care homes formed the basis of the review of the relevant literature. Given the timeliness and relevance of this review, with regard to the scope of the COS, it was not considered necessary to extend the literature review further. Twelve randomised controlled trials were included in the review, which collectively involved 10,953 older adults (65 years or older) resident in 355 care homes across ten countries. Studies included in the review tested the effect of a range of interventions which aimed to optimise care home residents’ complete medication regimens. The review did not include studies evaluating interventions aimed at specific medicines or medicine classes (e.g. benzodiazepines) or those concentrating on one healthcare condition. Further details on the literature search strategy and inclusion criteria have been reported in the review [22]. Information on every outcome measured and reported in each of the 12 trials was extracted verbatim and compiled in data extraction tables.

The second strategy employed was stakeholder involvement in the form of focus groups and semi-structured interviews. These were conducted as part of the wider CHIPPS programme; the primary aim of the interviews/focus groups was to define the components of the intervention (i.e. the service specification) in the CHIPPS trial. Stakeholder participants comprised general practitioners (GPs), pharmacists, care home managers, care homes staff and care home residents/relatives, located in four different sites across the UK. Recruitment to focus groups was achieved through several channels, beginning with Principal Investigators at each of the four sites identifying local gatekeepers for each stakeholder group. GPs, primary care and community pharmacists who had experience working in care homes were identified and approached via relevant local networks. Care homes were contacted initially using lists obtained from the relevant care home regulatory bodies’ websites, and then through existing local research networks. Participating care home managers then acted as gatekeepers to facilitate the recruitment of other care home staff, residents and relatives. Amongst those stakeholders who expressed an interest in participating, a purposive sampling approach was followed to maximise the diversity amongst these interested stakeholders. This approach helped to ensure a representative mix of stakeholders from urban and rural locations, multiple (chain) and independent care homes and large and small GP practices, for example. Participants unavailable to attend a stakeholder focus group were invited to participate in an individual interview. Whilst most of the topics covered in the interviews/focus groups centred on the CHIPPS intervention and pharmacist independent prescriber (PIP) training, participants were also asked what they viewed as ‘important outcomes to measure in studies which aimed to determine the effectiveness of interventions to optimise prescribing in care homes’.

Interviews and focus groups were audio-recorded and transcribed verbatim. Analysis of the transcripts was conducted independently by two researchers who extracted the outcomes proposed by the stakeholders verbatim.

Once all outcomes were identified and extracted from both the literature sources and the stakeholder focus groups and interviews, the resulting long-list of outcomes was reviewed and refined. Duplicate items were removed as were process measures, i.e. outcomes which are simply a measure of an aspect of the delivery of the intervention. In order to create a manageable list of outcomes for consideration in the Delphi exercise (see the following section), four members of the CHIPPS team (CH, DA, LS and RH), with expertise in the area (who were excluded from participation in the Delphi exercise) independently reviewed the list of outcomes and voted anonymously on whether they thought each outcome should be included in or excluded from the Delphi questionnaire. Only those outcomes which were voted by unanimous decision to be *excluded* were subsequently *omitted*.

### Phase 2: Delphi consensus exercise

The second phase of the COS development utilised a Delphi exercise to achieve consensus across the participant group on outcomes to be included in the final COS. The Delphi technique typically involves administering a series of rounds of questionnaires with anonymised feedback provided between each round.

As there are currently no guidelines concerning the ideal number of participants required to form a Delphi panel for COS development [13], it was decided, a priori, that the chosen panel would comprise the 19 members of the wider CHIPPS management team, all of whom had relevant aged care experience specifically relating to care homes. Recognising the importance of involving a wide range of stakeholders, the Delphi panel (*n* = 19) therefore was a heterogeneous group that included academic pharmacists (*n* = 3), geriatricians (*n* = 2), Patient Public Involvement (PPI) representatives (*n* = 2), health economists (*n* = 2), senior CHIPPS research fellows (*n* = 2), a prescribing advisor pharmacist (*n* = 1), an academic sociologist (*n* = 1), a research governance manager (*n* = 1), a care home quality director (*n* = 1), an educationalist (*n* = 1), an academic doctor (*n* = 1), a GP (*n* = 1) and an academic nurse (*n* = 1). As the panellists were based in disparate geographical locations across the UK, an online two-round Delphi questionnaire was administered to facilitate efficient data collection.

The refined list of outcomes generated during Phase 1 of the COS development process was used to construct a list of questionnaire items. At the start of the questionnaire, panellists were asked to rate how important they thought it was for each outcome to be included in a COS for studies involving optimising prescribing in care homes. Each questionnaire item was formatted with the outcome set out in bold font, and with a brief explanation of the outcome provided underneath, as in this example:

**Physical functioning**. Explanation: Care home residents’ physical functioning i.e. ability to perform physical tasks/everyday abilities (e.g. mobility, using stairs, dressing oneself, etc.)

The introduction to the questionnaire clarified for panellists that the ‘explanation’ should not be interpreted as an all-encompassing definition of the outcome, but rather as providing context and preventing misinterpretation of the meaning of the outcome terminology. Panellists were instructed to score the importance of each outcome on a Likert scale ranging from 1 to 9, where scores of 1 to 3 indicated an outcome of ‘limited importance’, 4 to 6 ‘important but not critical’ and 7 to 9 ‘critical’. This scoring system has been used widely by COS developers and is derived from the recommendations of the Grading of Recommendations Assessment, Development and Evaluation (GRADE) Working Group [25]. Furthermore, panellists were able to select ‘unable to score’ if they felt unable to offer an opinion on a particular outcome. At the end of the first round of the questionnaire, panellists were also invited to suggest additional outcomes which they considered to be important. Outcomes suggested by panellists were considered for inclusion in the second round of the questionnaire.

The Delphi panel was invited to complete the questionnaire via email; a link to the questionnaire on the SurveyGizmo® website was provided. All respondents of the first-round questionnaire were invited to participate in the second-round questionnaire. The second round included items for which no consensus had been reached in the first round (see ‘Data analysis’ section) and any new outcomes suggested by panellists in the first round. A personalised summary of the first-round scores (individual score, group mean score, group median score) for each outcome was sent with the email invitation for the second questionnaire round. The Delphi panel was instructed to consider the feedback provided whilst re-scoring the outcomes contained in the second-round questionnaire. For both rounds, reminder emails were sent as necessary to encourage participation, and a deadline of 4 weeks was given for completion.

### Data analysis

The Delphi survey responses were analysed using SPSS 22.0. For each outcome, the group mean and median scores were calculated for the feedback purposes described above. The round 1 questionnaires were analysed by calculating the percentage of participants rating each outcome as critically important (i.e. 7, 8 or 9) and not important (i.e. 1, 2 or 3). Consensus for an outcome being included in the COS was defined as ≥70% of respondents scoring an outcome 7–9 *and* <15% scoring the outcome 1–3. Conversely, consensus for an outcome being excluded from the COS was defined as ≥70% of respondents scoring an outcome 1–3 *and* <15% scoring the outcome 7–9. All other score distributions indicated that no consensus was achieved for the outcome [1]. Only those outcomes from round 1 for which no consensus was achieved following analysis of the round 1 results were retained for the round 2 questionnaire. Round 2 responses were analysed by applying the same consensus criteria as in round 1. Outcomes for which no consensus was achieved following the second round of the questionnaire were not included in the final COS.

In order to construct the final COS, the outcomes retained following both rounds of the Delphi exercise were organised under a hierarchy of outcome domains and categories [26] created by the grouping together of any closely related, overlapping but distinct individual outcomes. The classification of outcomes into domains and categories was achieved via discussion between three of the authors (AM, CH and DA).

## Results

In Phase 1, a total of 63 outcomes for potential inclusion in the COS were identified (22 from 12 studies included in the Cochrane systematic review and 41 from the stakeholder focus groups and interviews). The demography of the different stakeholders (*n* = 85) involved in identifying outcomes during the course of the interviews and focus groups is shown in Table 1.

**Table 1:** Stakeholder-specific focus groups and interviews

UK site	Number of focus groups	Participant type and numbers	Number of interviews	Participant type
A	3	Pharmacists × 4 GPs × 5 Residents/relatives × 8	2	Pharmacist × 1 GP × 1
B	3	Pharmacists × 8 GPs × 10 Care home staff × 2	4	GP × 1 Care home staff × 3
C	5	Pharmacists × 8 GPs × 7 Care home staff × 4 Care home managers × 3 Residents/relatives × 6	0	NA
D	2	Pharmacists × 5 GPs × 2	7	Pharmacist × 1 GPs × 3 Care home managers × 3
Total	13	72 participants	13	13 participants

**Fig. 1:**
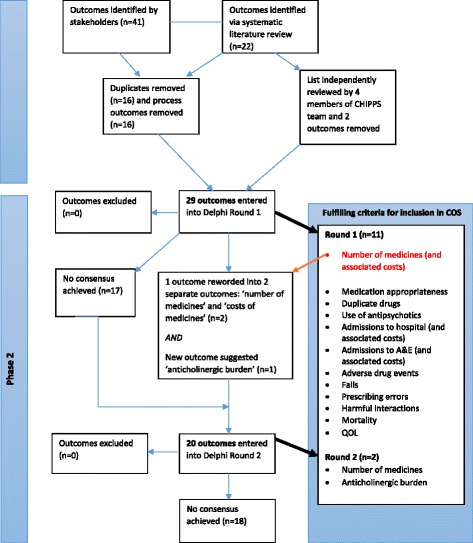
COS development overview

Of the 63 outcomes, 16 duplicates (i.e. outcomes which were identified in both the literature and also reported by the stakeholders) were removed as were 16 process measures. Examples of process measures that were removed included: ‘satisfaction with PIP service’ and ‘care home staff’s accuracy of record keeping’. Additionally, a unanimous decision was reached by the chosen four members of the CHIPPS team to exclude two outcomes (‘pain’ and ‘accidents’) from the Delphi exercise. This review and refinement therefore resulted in a total of 29 outcomes going forward to the first round of the Delphi questionnaire (see Fig. 1).

### Delphi consensus exercise

All 19 Delphi panellists completed the first round of the Delphi questionnaire (100.0% response rate). Following analysis, 12 outcomes met the consensus criteria for inclusion in the COS. No outcomes met the consensus criteria for exclusion from the COS; no consensus was achieved for 17 outcomes (see Table 2).

**Table 2:** Delphi questionnaire round 1 results

Outcome	Mean Delphi score	Median Delphi score	Respondents scoring 7–9 ‘critically important’ (%)	Respondents scoring 1–3 ‘not important’ (%)	Result (In, Out, No consensus)
Number of medications (and associated costs)	7.7	8.5	83.3	0	In
Medication wastage (and associated costs)	6.6	7	68.4	10.5	No consensus
Polypharmacy (≥4 medicines)	6.5	7	57.9	10.5	No consensus
Medication appropriateness (potentially inappropriate prescribing)	8.2	9	84.2	0	In
Duplicate drugs	7.2	7.5	72.2	5.6	In
Use of antipsychotics	7.4	8	73.7	0	In
Medication changes made (by anyone)	6.9	8	63.2	10.5	No consensus
Number of medication reviews conducted (by anyone)	6.7	7	63.2	10.5	No consensus
Admissions to hospital (and associated costs)	8.2	8	100	0	In
Accident and emergency (A&E) visits (and associated costs)	7.8	8	83.3	0	In
Visits to outpatients (and associated costs)	5.3	5	26.3	31.6	No consensus
Visits to/from GP (and associated costs)	7.1	7	63.2	5.3	No consensus
Visits to/from nurse (and associated costs)	6.1	6	42.1	5.3	No consensus
Adverse drug events	8.4	9	94.7	0	In
Falls	7.4	7	84.2	0	In
Acute kidney injury	6.7	6	46.7	0	No consensus
Prescribing errors	7.9	8	89.5	5.3	In
Harmful interactions	7.7	8	84.2	5.3	In
All-cause mortality	7.5	9	78.9	5.3	In
Physical functioning	6.5	7	57.9	15.8	No consensus
Behaviour	6.6	7	63.2	5.3	No consensus
Cognitive functioning	6.6	7	57.9	5.3	No consensus
Depression	6.3	7	55.6	5.6	No consensus
Quality of life	7.7	8	83.3	0	In
Compliance with NICE guidelines	6.3	7	52.6	10.5	No consensus
Compliance with medicines	6.7	7	68.4	5.3	No consensus
Care home staff job satisfaction	5	5	26.3	36.8	No consensus
Efficiency of medication administration by care home staff	6.3	6	42.1	5.3	No consensus
Accuracy of administration of medications by care home staff	6.9	7	57.9	5.3	No consensus

*NICE* National Institute for Health and Care Excellence

When asked if they wished to suggest any further outcomes that they thought should be included in the COS, in total, three participants suggested: ‘patient mobility’, ‘making sure drug charts are kept up to date’, ‘anticholinergic burden’, ‘nutritional status’, e.g. MUST (Malnutrition Universal Screening Tool) score, or ‘use of nutrition supplements’ and ‘appropriate use of covert medication’. Following discussion of these suggested items amongst three of the authors (AM, CH and DA), one new outcome (‘anticholinergic burden’) was added to the list for inclusion in the second round of the Delphi questionnaire. ‘Patient mobility’ was considered to be encompassed within the outcome ‘physical functioning’ and was not added. The other suggestions were not considered relevant for studies aiming to optimise *prescribing* (of medicines) in care homes and were therefore also not included.

In addition, feedback from one Delphi panel participant led to the re-formulation of the outcome ‘number of medicines (and associated costs)’ to two separate outcomes: ‘number of medicines’ and ‘costs of medicines’. As such, these two outcomes, along with anticholinergic burden and the 17 outcomes carried forward from round 1, resulted in a total of 20 outcomes being included in the second round of the Delphi questionnaire (see Fig. 1).

Eighteen of the 19 round 1 respondents completed round 2 (94.7% response). Two further outcomes (‘number of medicines’ and ‘anticholinergic burden’) met the criteria for consensus inclusion in the COS (see Table 3).

**Table 3:** Delphi questionnaire round 2 results

Outcome	Mean	Med	Respondents scoring 7–9 ‘critically important’ (%)	Respondents scoring 1–3 ‘not important’ (%)	Consensus Result (In, Out, No consensus)
Number of medications	7.3	8.0	83.3	11.1	In
Costs of prescribed medications	6.3	7.0	61.1	11.1	No consensus
Medication wastage (and associated costs)	6.6	7.0	66.7	5.6	No consensus
Polypharmacy (≥4 medicines)	6.6	7.0	66.7	5.6	No consensus
Medication changes made (by anyone	6.5	7.0	55.6	5.6	No consensus
Number of medication reviews conducted (by anyone)	6.6	7.0	66.7	5.6	No consensus
Visits to outpatients (and associated costs)	5.6	5.0	33.3	5.6	No consensus
Visits to/from GP (and associated costs)	6.6	6.5	50.0	0	No consensus
Visits to/from nurse (and associated costs)	6.1	6.5	50.0	0	No consensus
Acute kidney injury	6.8	7.0	53.3	0	No consensus
Physical functioning	6.5	7.0	61.1	5.6	No consensus
Behaviour	6.9	7.0	61.1	5.6	No consensus
Cognitive functioning	6.8	7.0	61.1	0	No consensus
Depression	6.7	7.0	61.1	0	No consensus
Compliance with NICE guidelines	6.4	7.0	55.6	16.7	No consensus
Compliance with medicines	6.9	7.5	61.1	5.6	No consensus
Care home staff job satisfaction	5.1	5.0	22.2	5.6	No consensus
Efficiency of medication administration by care home staff	6.4	6.0	38.9	0	No consensus
Accuracy of administration of medications by care home staff	7.3	7.0	55.6	0	No consensus
Anticholinergic burden	7.3	7.0	75.0	0	In

Therefore, a total of 13 individual outcomes met the criteria for inclusion in the COS following both rounds of the Delphi exercise. These 13 outcomes were grouped into a total of seven distinct outcome domains, which were then organised under three overarching categories of outcomes: medication-related, patient-related and healthcare utilisation-related outcomes (Table 4).

**Table 4:** Final COS for effectiveness studies in optimising prescribing in older adults in care homes

Category	Outcome domain • Outcome	Definition from Delphi questionnaire
Medication-related	1. Medication appropriateness (potentially inappropriate prescribing)	Potentially inappropriate prescribing ‘encompasses the use of medicines that introduce a significant risk of an adverse drug-related event where there is evidence for an equally or more effective but lower-risk alternative therapy available for treating the same condition…also includes the use of medicines at a higher frequency and for longer than clinically indicated, the use of multiple medicines that have recognised drug-drug interactions and drug-disease interactions, and importantly, the under-use of beneficial medicines that are clinically indicated but not prescribed for ageist or irrational reasons’ [53]
	• Number of prescribed medicines	Number of medications prescribed for a care home resident
	• Duplicate drugs	’Duplicate drugs’ described a situation where an individual is prescribed two medicines of the same pharmacological class, e.g. the prescribing of two concurrent opiates [54]
	• Use of antipsychotics	The prescription of antipsychotic medicines in care home residents. ‘Antipsychotic drugs are also known as “neuroleptics” and (misleadingly) as “major tranquillisers”. In the short term they are used to calm disturbed patients whatever the underlying psychopathology… The balance of risks and benefits should be considered before prescribing antipsychotic drugs for elderly patients’ [55]
	• Harmful interactions	A ‘harmful interaction’ in a care home resident may describe the prescription of a medication which causes or has the potential to cause a clinically significant drug-drug or drug-disease interaction. A drug-drug interaction is when a medicine affects the pharmacological effect of another medicine. A drug-disease interaction is when a medicine, which may be used to treat or prevent one disease, can have a detrimental effect on another existing disease/condition in the individual [56]
	• Anticholinergic burden	The anticholinergic burden associated with care home residents’ medication regimens. Medicines with anticholinergic effects are commonly prescribed for various conditions; however, increased overall exposure to anticholinergics has been associated with an increased risk of cognitive impairment, falls and all-cause mortality in older adults [57]
	2. Adverse drug events	Adverse drug events experienced by care home residents. ‘An adverse drug event is any undesirable event experienced by a patient whilst taking a medicine, including physical harm, mental harm, or loss of function’ [58]
	3. Prescribing errors	Prescribing errors in care home residents’ medication regimens. A prescribing error is ‘a prescribing decision that results in an unintentional, significant: (1 reduction in the probability of treatment being timely and effective, or (2 Increase in the risk of harm, when compared to generally accepted practice’ [59]
Patient-related	4. Falls	Falls occurring amongst care home residents. A fall is ‘an event which results in a person coming to rest inadvertently on the ground or floor or other lower level’ [60]
	5. Quality of life	A measure of care home residents’ quality of life (QoL). QoL is ‘a ubiquitous concept that has different philosophical, political and health-related definitions. Health-related QoL includes the physical, functional, social and emotional well-being of an individual’ [61]
	6. All-cause mortality	All deaths of care home residents
Healthcare utililisation-related	7. Admissions to hospital (and associated costs)	The number of care home residents having a hospital admission/number of hospital admissions per resident (and the associated costs)
	• Accident and emergency (A&E) visits to hospital (and associated costs)	The number of care home residents attending A&E departments/number of A&E visits per resident (and the associated costs)

## Discussion

This study used a comprehensive approach involving an up-to-date systematic literature review, stakeholder involvement and formal consensus methodology to develop a COS for effectiveness studies aimed at optimising prescribing in older adults in care homes. The final COS comprises 13 outcomes, arranged under seven broader outcome domains: medication appropriateness (potentially inappropriate prescribing), adverse drug events, prescribing errors, falls, quality of life, all-cause mortality and admissions to hospital (and associated costs). It is recommended that this COS be used to guide outcome selection in future studies conducted in this research area.

The outcomes contained in this COS have been derived from both the published literature and the input of a large, wide-ranging number of relevant stakeholders, including healthcare professionals and service users/their relatives. Furthermore, the outcomes selected for inclusion in the final COS have been rated as critically important by a panel of experts in the field. It is evident that there is a relationship between the seven overarching outcome domains that comprise this COS. Inappropriate prescribing of medicines, which may be the result of errors in prescribing [27], is known to be associated with an increased risk of adverse drug events (ADEs) [28]. Furthermore, ADEs are frequently a causative or contributory factor to hospitalisation in older adults [29]. Anticholinergic burden, a measure of a patient’s cumulative exposure to medicines with anticholinergic properties, has been shown to be associated with an increased risk of hospitalisation for confusion or dementia [30]. Falls, which may also be a manifestation of an ADE, are of particular concern amongst institutionalised older adults, as they are directly responsible for significant morbidity and mortality in this population [31]. Additionally, quality of life (QoL) is an important target for interventions, as the loss of personal independence and the frailty which characterises the care home population means it is an outcome of particular significance [32]. Whilst interventions to optimise prescribing often are primarily focussed on improving healthcare system-related outcomes (e.g. hospitalisations), it is important that researchers in the future also focus on patient-centred outcomes, including QoL.

This study has several strengths in its design and conduct. We have followed the guidelines for COS development, as outlined by the COMET Initiative [1]. This study used multiple comprehensive approaches to identify outcomes for potential inclusion in the COS. Only those studies with randomised controlled designs were included in the systematic review that was subsequently used to generate the long-list of outcomes. It is therefore possible that had the literature search criteria been expanded to include other study designs, further outcomes may have been identified for potential inclusion in the COS. However, the review was not the sole means of identifying potential outcomes, as opportunities were provided for stakeholders to suggest outcomes of importance to them. Relevant stakeholders, including care home residents and their relatives as well as healthcare professionals, were actively involved in identifying outcomes of importance to them. Involving service users and/or their representatives is encouraged in the field of COS development, as these individuals may identify outcomes of importance that may not otherwise be identified in the published literature or by other stakeholders, such as healthcare professionals [13, 33, 34]. Furthermore, the relative importance placed on various outcomes can often differ between patients, service users and healthcare professionals [35, 36]. The inclusion of key stakeholder groups throughout the development of this COS was an intentional effort to maximise its relevance to these groups and therefore its acceptance in future research.

This study employed a well-established and widely used method of achieving consensus in order to facilitate the inclusion of a broad range of panel participants in geographically diverse locations. The Delphi consensus technique has been frequently employed by other COS developers as a means of reaching consensus [37–39]. As individuals participating in the Delphi exercise need not directly interact with each other, this consensus technique has the advantage of preventing bias resulting from more vocal or senior panellists dominating the views of the group, which is more likely in a face-to-face setting [36]. Furthermore, the online administration of Delphi questionnaires avoids the logistical, practical and economic challenges typically associated with postal questionnaires or face-to-face meetings [40]. Therefore, whilst this consensus method was considered appropriate for use for the above reasons, an alternative method may have led to a different final set of outcomes. The response rates for the questionnaire were 100% and 94.7% for rounds 1 and 2, respectively. This low rate of attrition may reflect the composition of the Delphi panel from members of the wider study team.

The main limitation of this study is that all the Delphi panel participants were from the UK, which may affect the wider generalisability of the results. It is possible that a larger, international Delphi panel may have produced a different COS, as certain outcomes could be valued differently in other countries. The widespread adoption and reporting of outcomes contained in this COS in future studies would aid in its validation outside of the UK setting. Another possible limitation is that there were no pre-specified proportions of the various groups of individuals (i.e. doctors, PPI representatives, etc.) who comprised the Delphi panel. Previous COS developers have recruited patients and professionals to their Delphi panels in a 2:1 ratio, to give preference to patient-reported outcomes [37, 41]. Therefore, it is possible that some outcomes may not have met the consensus criteria for inclusion as a result of under-representation of a particular stakeholder group.

Unlike previous COS developers [37, 39, 41], we did not implement a ‘consensus meeting’ following the Delphi exercise. The decision not to include a consensus meeting in the COS development process was a pragmatic one. Holding a final consensus meeting to make decisions on whether the ‘undecided’ outcomes should be included in the final COS may have resulted in a larger final COS. However, it should be noted that consensus had been reached for 13 outcomes (seven outcome domains) following the two planned rounds of the Delphi. It is important to bear in mind that a COS represents a *minimum* number of outcomes that, ideally, should be measured in all trials in a specific area; additional outcomes can be freely included if deemed relevant [4].

In this study, outcomes that had not achieved consensus support following the second round of the Delphi were excluded from the COS, despite not having met the pre-defined consensus criteria for exclusion. It is possible that further Delphi rounds may have achieved consensus on more outcomes; however, due to time restraints and a pre-defined Delphi of two rounds, this was not possible. There are currently no guidelines for the most appropriate number of Delphi rounds to be conducted. It is therefore recommended that future COS developers give consideration as to how they will deal with ‘undecided outcomes’ following the final round of a Delphi questionnaire, should they choose not to include a final face-to-face consensus meeting as part of the process. It may also be worthwhile to ask participants to provide a rationale for each of their scores in the first round. Such information could then be collated and presented alongside the group score feedback to all participants, in order to inform individuals’ judgements in the second round of scoring the outcomes, thus facilitating group consensus.

It is important to note that the proposed COS has been developed to guide researchers on *what* to measure. The COS does not indicate *how* (or when) to measure and report these outcomes in a study. Of the 12 studies included in the systematic review from which outcomes were identified for potential inclusion in this COS, five studies measured medication appropriateness as an outcome. Across these five studies alone, three different tools were used to measure ‘medication appropriateness’ as an outcome: Screening Tool of Older People’s Prescriptions/Screening Tool to Alert doctors to Right Treatment (STOPP/START) [42], the Medication Appropriateness Index (MAI) [43] and a modified version of the Beers Criteria [44]. Similarly, in the two papers included in the review that measured and reported QoL as an outcome, two different measurement tools were used: the 15D [45] and the 12-Item Short-Form Health Survey (SF-12) [46]. Many more measurement tools exist to measure both these, and other, outcomes, including those outcomes suggested by stakeholders, such as ‘anticholinergic burden’, which can be quantified with a number of different tools including the Drug Burden Index [47] and the Anticholinergic Cognitive Burden Scale [48]. A detailed review or recommendation on the instruments to be used to measure the outcomes included in this COS, including medication appropriateness, QoL and anticholinergic burden, is beyond the scope of this paper and a current limitation of COSs in general.

Nonetheless, determining the most appropriate methods of measuring the outcomes in this COS will be crucial for its widespread acceptance and usefulness. In order to reduce heterogeneity in outcome measurement between trials, the next step will therefore be to determine how outcomes included in this COS should ideally be measured. Guidelines on how to select outcome measurement instruments are currently being developed by the Core Outcome Measurement Instrument Selection (COMIS) project group [49]. It is possible that this COS may highlight the absence of appropriate outcome measurement instruments, either because no instrument exists to measure the outcome or because the evidence base for existing measurement instruments is of limited quality. Furthermore, where several instruments all purporting to measure the same outcome exist, it may not be clear which instrument would be most appropriate. The COSMIN (Consensus-based Standards for the selection of health Measurement Instruments) checklist can be used to evaluate the methodological quality of studies on measurement properties (e.g. validity and reliability) and therefore may also be used to help inform the selection of the most appropriate measurement instrument for an outcome [50]. Furthermore, measurement properties including the acceptability of the measure (e.g. for patients and healthcare professionals) and its responsiveness to clinical change (i.e. its sensitivity to detect meaningful change) are also important considerations when selecting a measurement instrument [51].

In line with recommendations from COMET, this COS should be subject to review in the future as a form of validation to ensure outcomes are still relevant and important. Such reviews should also facilitate the addition of new outcomes, where necessary, through emergent evidence and the engagement of further key stakeholders [52].

## Conclusions

This work has identified a list of 13 outcomes (categorised into seven core outcome domains) to be measured and reported as a minimum in effectiveness studies aimed at optimising prescribing in older adults in care homes. To the authors’ best knowledge, this is the first COS for effectiveness studies in this field. It is recommended that this COS be reviewed periodically to validate the continued importance and relevance of its outcomes and to allow new outcomes to be added when necessary [1]. The next step will be to determine how best to measure outcomes included in this COS, so as to facilitate evidence synthesis by reducing heterogeneity in outcome measurement between future trials in this area.

## Abbreviations

A&E: Accident and emergency
AIDS: Acquired immunodeficiency syndrome
CHIPPS: Care Homes Independent Pharmacist Prescribing Study
COMET: Core Outcome Measures in Effectiveness Trials
COMIS: Core Outcome Measurement Instrument Selection
COS: Core outcome set
COSMIN: Consensus-based Standards for the selection of health Measurement Instruments
GP: General practitioner
HIV: Human immunodeficiency virus
MUST: Malnutrition Universal Screening Tool
NICE: National Institute for Health and Care Excellence
ORBIT: Outcome Reporting Bias in Trials
PIP: Pharmacist independent prescriber
PPI: Patient Public Involvement
QoL: Quality of life
UK: United Kingdom
